# Annealed Polyvinyl Alcohol Hydrogels for Cartilage Replacement: Effects of Synthesis Parameters on Mechanical Properties

**DOI:** 10.3390/gels11080644

**Published:** 2025-08-14

**Authors:** Hassan Mahmoud, Christian M. Puttlitz, Benjamin C. Gadomski, Kevin M. Labus

**Affiliations:** 1 School of Biomedical Engineering, Colorado State University, Fort Collins, CO 80523, USA; 2 Department of Mechanical Engineering, Colorado State University, Fort Collins, CO 80523, USA; 3 Department of Clinical Sciences, Colorado State University, Fort Collins, CO 80523, USA

**Keywords:** polyvinyl alcohol (PVA), annealing, molecular weight (MW), articular cartilage, mechanical properties

## Abstract

The objective of this paper was to determine the interactive effects of multiple synthesis parameters on annealed PVA hydrogel properties and assess these hydrogels for the application of cartilage replacement. PVA hydrogels were synthesized at two different molecular weight ranges (89–98 kDa and 146–186 kDa), two polymer concentrations (10% PVA and 20% PVA), and four different annealing temperatures (120 °C, 135 °C, 150 °C, and 165 °C). The compressive, tensile, and wear mechanical properties were measured, and the crystalline structure of these hydrogels was assessed via differential scanning calorimetry. Hydrogels showed increasing polymer weight percent, tensile modulus, and compressive modulus with increasing annealing temperature. Depending on synthesis parameters, the hydrogels matched or exceeded the previously published compressive and tensile properties of native cartilage. Higher molecular weight PVA hydrogels (146–186 kDa) exhibited less wear, but greater friction, compared to lower molecular weight PVA (89–98 kDa). The PVA hydrogels exhibited crystallinity in the range of 53–78%, but no consistent differences in crystallinity were detected between hydrogel variants. It was concluded that the (10% PVA, 146 kDa, 165 °C) annealed PVA hydrogel demonstrated the most appropriate balance of high tensile strength and compressive compliance comparable to cartilage.

## 1. Introduction

The main function of articular cartilage is to dissipate joint contact stresses and allow for low-friction movement between contacting bone surfaces. However, articular cartilage is highly susceptible to damage, which can lead to joint dysfunction, tissue degradation, and the development of osteoarthritis [1]. Common treatment methods for cartilage defect repair include microfracture, autologous chondrocyte implantation, and osteochondral allograft implantation [2,3]. However, articular cartilage has a limited ability to repair or regenerate due to the lack of lymphatics, nerves, or blood vessels, making it difficult to receive oxygen and nutrients [2]. Therefore, these treatments often fail to repair the cartilage tissue or treat the symptoms. For cases with low regenerative potential, artificial cartilage materials may be preferred for local defect repair [3,4,5,6].

Polyvinyl alcohol (PVA) hydrogels have been proposed as an artificial cartilage material [5]. PVA hydrogels exhibit many properties that are beneficial for biomedical applications, such as cartilage replacement, including the ability to absorb and retain water, biocompatibility, and chemical stability [7]. PVA hydrogels are hydrophilic and allow for the passage of nutrients and waste, making them more tolerated by the human body. In addition to this, their ability to absorb and retain water further helps to promote diffusion and reduce inflammation [8]. PVA hydrogels and cartilage exhibit similar permeability and viscoelasticity [5,6], distinguishing PVA as a promising material choice for cartilage repair [7,9].

The mechanical properties of PVA hydrogels can be modified by annealing to meet the high strength requirements for articular cartilage replacement [6]. PVA hydrogels are hydrophilic due to hydrogen bonds created between water molecules and free hydroxyl groups within the hydrogel. When annealing a PVA hydrogel, the free hydroxyl groups within the polymer form bonds with each other due to a movement of the physical cross-links of the hydrogel, resulting in increased mechanical properties associated with increased cross-linking, increased crystallinity, and decreased water content [6]. Further, PVA molecular weight (MW), polymer concentration, and annealing temperature can all impact PVA hydrogel properties [7]. Increasing the MW of a polymer can lead to greater tensile strength and impact resistance. At the molecular scale, this translates to more polymer chains, which have an increased probability of entangling with one another to create a higher degree of interconnectedness within the polymer [10]. Increasing the polymer concentration of a hydrogel can lead to increases in elastic modulus and compressive strength, creating a denser structure that is more resistant to deformation [11]. Tuning the annealing temperature of a polymer has been shown to increase polymer crystallinity, thereby leading to a more organized structure of polymer chains and a mechanically stronger hydrogel [12]. The way in which these synthesis parameters interact with one another to affect hydrogel properties has not been fully defined in the literature. The purpose of this study was to analyze the interactive effects of MW, polymer concentration, and annealing temperature on the structure and mechanical properties of annealed PVA hydrogels and to assess the biomimetic properties of these hydrogels for the application of articular cartilage replacement.

**Figure 1:**
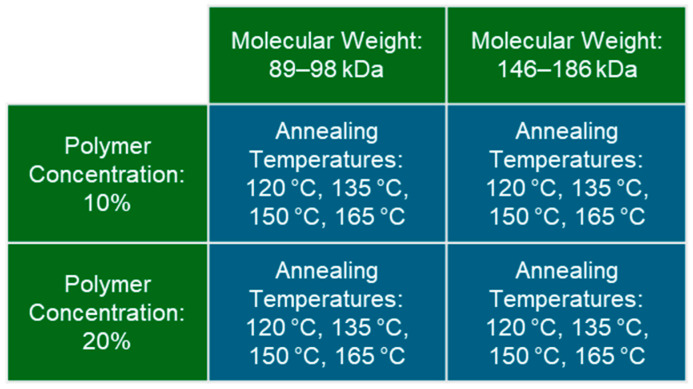
Summary of the testing groups in the full factorial study design consisting of two molecular weights, two polymer concentrations, and four annealing temperatures.

This study included a full factorial combination of PVA hydrogels across 2 MWs, 2 polymer concentrations, and 4 annealing temperatures, totaling 16 unique hydrogel variants (Figure 1). MWs of 89–98 kDa and 146–186 kDa were selected as relatively medium and high MWs for PVA, and prior studies have shown higher MWs to exhibit stronger mechanical properties [7]. Polymer concentrations of 10% and 20% were selected because preliminary studies and prior research have demonstrated consistent hydrogels and high mechanical properties following annealing for this range of polymer concentration [7]. PVA hydrogels are annealed between the glass transition temperature of about 90 °C and the melting temperature of about 230 °C for fully hydrolyzed PVA. Annealing temperatures were selected in the middle of this range (Figure 1) because our preliminary studies demonstrated softer hydrogels at lower annealing temperatures and brittle hydrogels at higher annealing temperatures. The hydrogels were analyzed via polymer weight percent measurements, unconfined compression testing, tensile testing, cyclic wear testing, and differential scanning calorimetry (DSC).

## 2. Results and Discussion

### 2.1. Polymer Weight Percent

**Figure 2:**
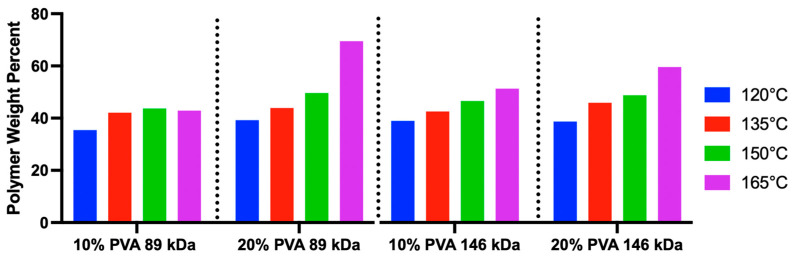
Results of the polymer weight percent for each PVA hydrogel variant (*n* = 1 hydrogel was measured per group).

Increasing annealing temperature was significantly correlated (linear regression, *p* = 0.0005, R^2^ = 0.59) with increased polymer weight percent (*wt%*) across all MWs and polymer concentrations (Figure 2). No consistent effects were observed for MW or polymer concentration on the hydrogels’ polymer *wt%*.

### 2.2. Unconfined Compression

**Figure 3:**
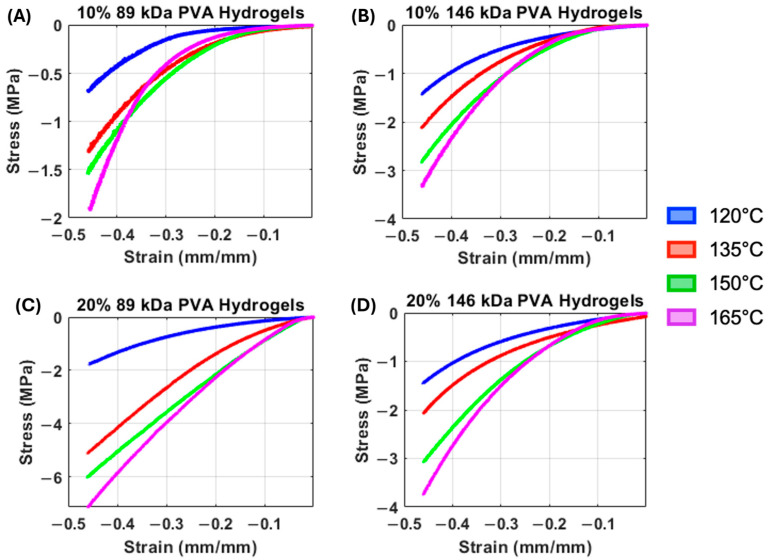
(**A**) Median compressive stress–strain curves of the 10% 89 kDa PVA hydrogels. (**B**) Median compressive stress–strain curves of the 10% 146 kDa PVA hydrogels. (**C**) Median compressive stress–strain curves of the 20% 89 kDa PVA hydrogels. (**D**) Median compressive stress–strain curves of the 20% 146 kDa PVA hydrogels.

**Figure 4:**
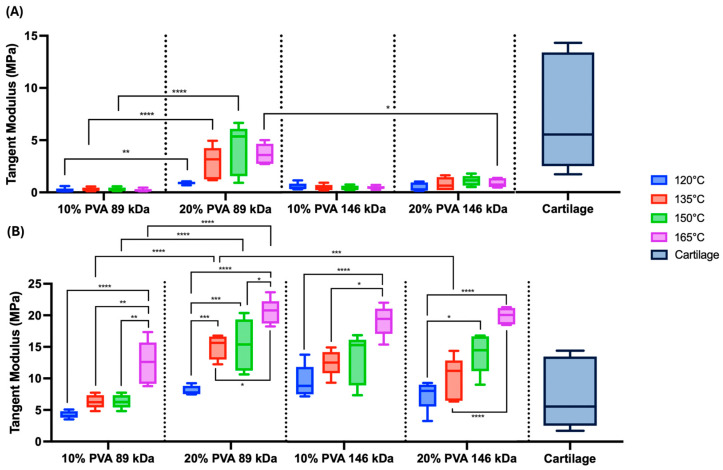
Unconfined compression testing results. (**A**) Summary data of the low-strain tangent moduli. Main effects on low-strain tangent moduli: annealing temperature (*p* = 0.006), MW (*p* = 0.06), and polymer concentration (*p* < 0.0001). (**B**) Summary data of the high-strain tangent moduli. Main effects on high-strain tangent moduli: annealing temperature (*p* < 0.0001), MW (*p* = 0.35), and polymer concentration (*p* < 0.0001). Data are shown in standard box-and-whisker format. Statistical comparisons are shown across annealing temperatures within MW and polymer concentration groups as well as across MW and polymer concentration within each annealing temperature (* *p* < 0.05, ** *p* < 0.006, *** *p* < 0.0008, & **** *p* < 0.0001). Cartilage compressive modulus data are from prior published studies [13,14,15]. A sample size of *n* = 5 was tested for each hydrogel group.

The median representative compressive stress–strain curves of PVA hydrogels demonstrate a general trend of increasing stiffness with increasing annealing temperature (Figure 3). Accordingly, there was a statistically significant overall main effect of annealing temperature on both low-strain tangent moduli (Figure 4A, *p* = 0.006) and high-strain tangent moduli (Figure 4B, *p* < 0.0001), with increasing temperature resulting in increasing compressive moduli. However, there was also a significant interaction effect between annealing temperature and polymer concentration for low-strain tangent moduli (*p* = 0.04), and annealing temperature did not significantly impact the low-strain tangent modulus in 10% PVA groups.

All compressive stress–strain curves demonstrated nonlinear behavior (Figure 3). This nonlinearity appears to be more pronounced in the 10% PVA, 89 kDa hydrogels at all annealing temperatures, with each curve demonstrating a low stiffness at low-strain magnitudes (Figure 3A). Accordingly, the low-strain tangent modulus exhibited a significant interaction effect between MW and polymer concentration (*p* < 0.0001), where the (20% PVA, 89 kDa) group exhibited the greatest low-strain tangent modulus, followed by (20% PVA, 146 kDa), (10% PVA, 146 kDa), then (10% PVA, 89 kDa). There was also a statistically significant main effect of polymer concentration, with 20% PVA groups exhibiting greater low-strain tangent moduli compared to 10% PVA groups (*p* < 0.0001).

The high-strain tangent modulus also exhibited a significant main effect of polymer concentration, with 20% PVA groups exhibiting greater moduli compared to 10% PVA groups (*p* < 0.0001). There were significant interaction effects on high-strain modulus between MW and polymer concentration (*p* < 0.0001). Specifically, within the 89 kDa MW groups and across equivalent annealing temperatures, the 20% PVA groups exhibited high-strain tangent moduli compared to 10% PVA groups (Figure 4B). Furthermore, the (20% PVA, 89 kDa) hydrogels exhibited high-strain tangent moduli compared to the higher molecular weight (20% PVA, 146 kDa) group.

The hydrogel compressive results were compared to published compressive moduli of articular cartilage, obtained via unconfined compression under various loading rates and magnitudes [13,14,15]. The PVA hydrogels exhibited low-strain moduli that were generally at the low end of the range of cartilage compressive moduli (Figure 4A). Depending on annealing temperature, the high-strain moduli of PVA hydrogels were within the range or exceeded the range of cartilage compressive moduli (Figure 4B).

### 2.3. Tensile Testing

**Figure 5:**
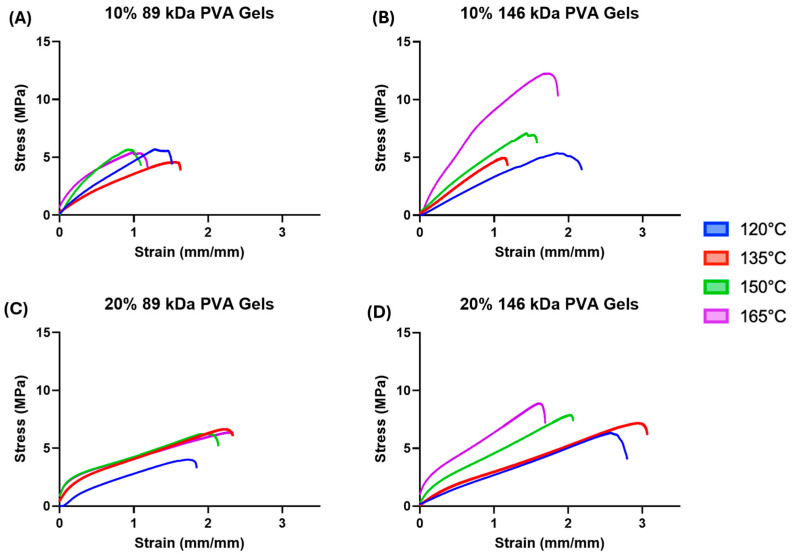
(**A**) Median tensile stress–strain curves of the 10% 89 kDa PVA hydrogels. (**B**) Median tensile stress–strain curves of the 10% 146 kDa PVA hydrogels. (**C**) Median tensile stress–strain curves of the 20% 89 kDa PVA hydrogels. (**D**) Median tensile stress–strain curves of the 20% 146 kDa PVA hydrogels.

Median tensile stress–strain curves are shown in Figure 5. The 10% PVA groups (Figure 5A,B) appear to exhibit relatively linear elastic stress–strain behavior when compared to the 20% PVA groups (Figure 5C,D). These groups demonstrated an initial linear elastic behavior, followed by a distinct yield point, and a second linear phase with a decreased slope. Although there are some noticeable differences in failure strains between groups, all PVA hydrogel variants exhibit failure strains much greater than the expected strains for cartilage replacement applications.

**Figure 6:**
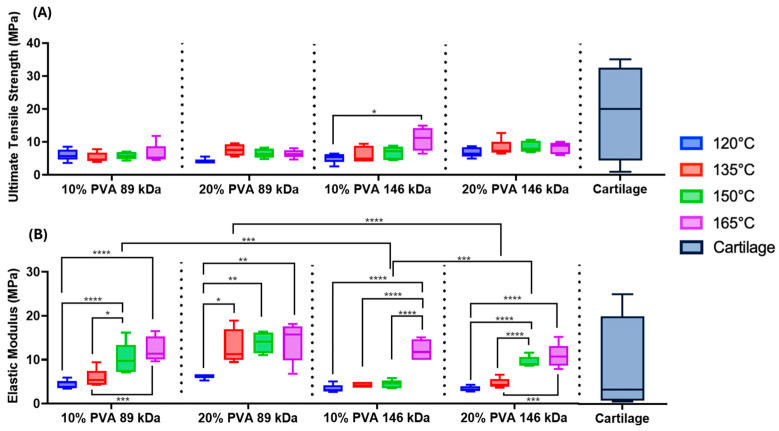
(**A**) Summary data of the ultimate tensile strength of each PVA hydrogel. Main effects on tensile strength: annealing temperature (*p* = 0.01), MW (*p* = 0.004), and polymer concentration (*p* = 0.10). (**B**) Summary data of the tensile elastic modulus of each PVA hydrogel. Main effects on tensile modulus: annealing temperature (*p* < 0.0001), MW (*p* < 0.0001), and polymer concentration (*p* < 0.0001). Statistical comparisons are shown across annealing temperatures within MW and polymer concentration groups as well as across MW and polymer concentration within each annealing temperature (* *p* < 0.05, ** *p* < 0.003, *** *p* < 0.0005, & **** *p* < 0.0001). Cartilage data are from prior published studies [13,16,17,18]. A sample size of *n* = 5 was tested for each hydrogel group.

Across all combinations of molecular weight and polymer concentration, increasing annealing temperature corresponded to a trend of increasing tensile modulus (Figure 6B, annealing temperature main effect: *p* < 0.0001); however, the effect of annealing temperature on tensile strength was less consistent (Figure 6A). The 10% PVA, 146 kDa hydrogels annealed at 165 °C exhibited the greatest mean tensile strength (10.89 ± 3.59 MPa) of all hydrogel variants.

There were significant main effects of MW on tensile properties. The 146 kDa MW group exhibited a 22% greater mean tensile strength compared to the 89 kDa MW group (*p* = 0.004). However, the 89 kDa MW group exhibited a 57% greater mean tensile modulus compared to the 146 kDa MW group (*p* < 0.0001). Hydrogels with a 20% polymer concentration exhibited a 58% greater tensile modulus compared to hydrogels with a 10% polymer concentration (*p* < 0.0001).

The hydrogel variants were within the lower range of strength of the published cartilage values, but were not quite able to match the maximal strength of articular cartilage [16]. Depending on annealing temperature, the PVA hydrogels fell within the range of previously reported tensile modulus values for cartilage [13,16,17,18].

### 2.4. Wear Testing

**Figure 7:**
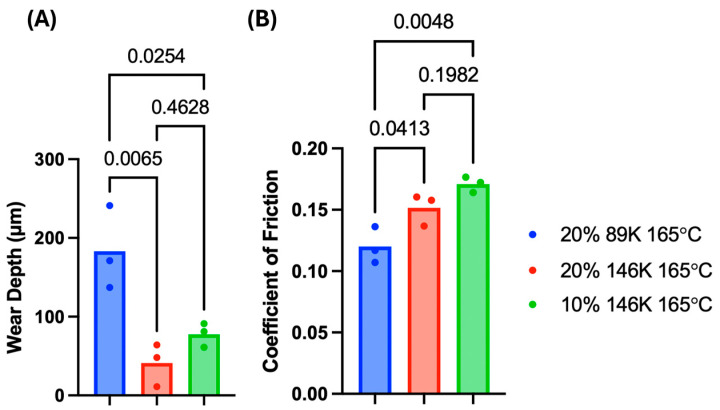
The (**A**) wear depths and (**B**) COF values from wear testing PVA hydrogels; *p*-values are shown for each statistical group comparison. A sample size of *n* = 3 was tested for each hydrogel group.

Following 500,000 cycles of wear on selected hydrogel variants, the mean wear depth of the (20% PVA, 89 kDa, 165 °C), (10% PVA, 146 kDa, 165 °C), and (20% PVA, 146 kDa, 165 °C) hydrogels were 0.18 mm, 0.078 mm, and 0.041 mm, respectively. The higher MW hydrogels (10% PVA, 146 kDa, 165 °C) and (20% PVA, 146 kDa, 165 °C) showed similar wear depths, but both higher MW hydrogels exhibited significantly less wear compared to the lower MW (20% PVA, 89 kDa, 165 °C) hydrogels (Figure 7A).

The mean of the COF values of the (20% PVA, 89 kDa, 165 °C), (10% PVA, 146 kDa, 165 °C), and (20% PVA, 146 kDa, 165 °C) hydrogels were 0.120, 0.171, and 0.152, respectively. The COF of the (20% PVA, 89 kDa, 165 °C) hydrogel was significantly lower than that of the (10% PVA, 146 kDa, 165 °C) and (20% PVA, 146 kDa, 165 °C) hydrogels (Figure 7B). These results are indicative of a primary effect of MW, with the higher MW hydrogels exhibiting less wear but greater friction.

### 2.5. Differential Scanning Calorimetry

**Figure 8:**
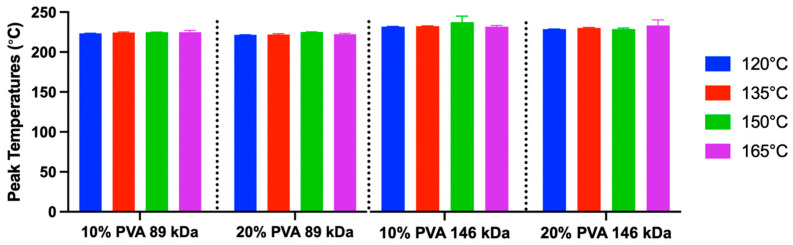
Peak melting temperature data derived from DSC (mean and standard deviation). A sample size of *n* = 3 was used for each group. Main effects on peak temperature: annealing temperature (*p* = 0.14), MW (*p* = 0.17), and polymer concentration (*p* = 0.45).

There were no significant differences in melting temperature between hydrogel groups (Figure 8). The overall mean melting temperature was 223.10 °C, and the range was 218.83–232.83 °C.

**Figure 9:**
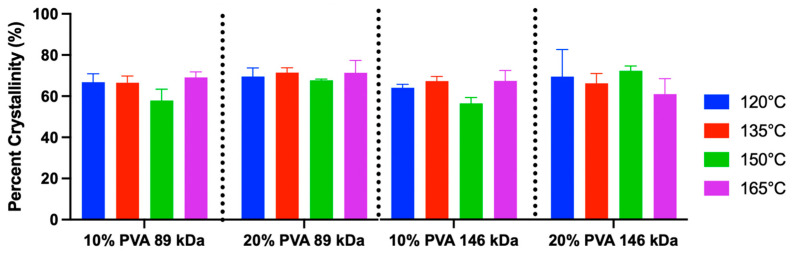
Crystallinity percentage of each hydrogel variant derived from DSC (mean and standard deviation). Main effects on crystallinity: annealing temperature (*p* = 0.22), MW (*p* = 0.20), and polymer concentration (*p* = 0.01). A sample size of *n* = 3 was used for each group.

The crystallinity calculations from DSC curves demonstrated a range of 53–78% crystallinity (Figure 9). The mean crystallinity across all groups was 64%. There was a significant main effect of polymer concentration. Specifically, the 20% PVA group exhibited a 4% greater mean crystallinity compared to the 10% PVA group (*p* = 0.01). There were no significant effects of annealing temperature (*p* = 0.22) or MW (*p* = 0.20) on crystallinity.

### 2.6. Effects of Annealing Temperature

The purpose of these experiments was to broadly characterize the effects of multiple synthesis parameters on the properties of PVA hydrogels for the application of cartilage replacement. The effects of annealing temperature were most prominent in this study. Annealing PVA hydrogels has been previously shown to greatly impact the hydrogel structure and properties [7,19]. Specifically, PVA hydrogels heated to temperatures above the glass transition temperature undergo a reorganization of the amorphous regions, which is characterized by increased physical cross-linking and crystallization [7]. This is facilitated by first drying the hydrogels, which removes the bonded water molecules, freeing the hydroxyl groups of the PVA chains to form cross-links via hydrogen bonds [20]. The thermal energy during annealing encouraged chain movement to increase the development of these physical cross-links, which resulted in fewer available hydroxyl groups that were free to bond with water, decreasing water uptake. Accordingly, the annealed PVA hydrogels exhibited a polymer weight percent that was approximately 2–5 times greater than the polymer concentration of the original PVA solution. This was confirmed via linear regression, which demonstrated that increasing annealing temperature was correlated with an increased polymer weight percent. This affected the mechanical properties as well. Specifically, the PVA hydrogels exhibited increasing tensile and compressive moduli with increasing annealing temperature. However, there was no clear effect of annealing temperature on PVA crystallinity. Therefore, increased mechanical properties at high annealing temperatures are more likely due to the greater cross-linking of the amorphous regions.

Previous studies that used DSC to determine the crystallinity of annealed PVA hydrogels found that increasing annealing temperature led to increased crystallinity. These papers found a range of crystallinity values between 23 and 71% [7,19,21]. The results of the current study did not demonstrate a similar effect of annealing temperature on crystallinity via DSC. However, the crystallinity range in this study (53–78%) was at the upper range of those found in prior studies. Additionally, the annealing temperature range of 120–165 °C was higher than that of the published experiments (90–135 °C) [19]. These results suggest that the annealing procedure used in the current study may have maximized the potential crystallinity of the material, regardless of the annealing temperatures. Annealing time may have also influenced these results. The total time spent annealing in the prior study ranged from 30 to 90 min [19], whereas a total time of 4 h was used in the current study; annealing time was not parameterized in this study.

### 2.7. Effects of Polymer Concentration

The PVA polymer concentration had a moderate impact on compressive modulus, with the 20% PVA group exhibiting low-stain and high-strain mean tangent moduli 84% and 58% greater than the 10% PVA group, respectively. The polymer concentration appeared to have an effect on the tensile modulus of the hydrogels (20% PVA hydrogels were 58% stiffer than 10% PVA hydrogels), but not on the hydrogels’ tensile strength. Additionally, the 20% PVA hydrogels were 4% more crystalline than the 10% PVA hydrogels. While this difference in crystallinity was minor, it may have impacted tensile and compressive moduli. Polymer concentration did not have a significant effect on friction or wear properties. Polymer concentration during PVA solution mixing has been previously shown to directly and substantially affect PVA hydrogel mechanical properties when synthesized using a freeze–thaw process [22]. However, in the current study, these differences may have been minimized by the annealing process, which increased the polymer weight percent of the final hydrogels. No consistent differences were seen in polymer weight percent between the 10% PVA and 20% PVA hydrogels.

### 2.8. Effects of Molecular Weight

The PVA MW had a moderate effect on hydrogel properties. The higher MW group (146–186 kDa) exhibited significantly greater tensile strength (ANOVA main effect), greater resistance to wear, lower tensile moduli (ANOVA main effects), and greater friction compared to the lower MW (89–98 kDa). There was no significant effect of the MW on the hydrogel’s crystallinity. These results indicate that the higher MW may contribute to a more durable material with greater strength and wear resistance, while also exhibiting a lower elastic modulus that better approximates that of native cartilage. These results are likely attributable to the greater polymer chain entanglement. Longer polymer chains in the higher MW material can lead to stronger, yet more compliant, polymers [7,20]. The shorter polymer chains in the 89 kDa hydrogels may allow more polymer chain mobility, enabling more physical cross-links to form and contributing to the higher tensile moduli observed. Similarly, the significantly lower wear depth in the 146 kDa hydrogels is consistent with previous studies showing that the greater chain entanglement in polymers with higher MW results in better resistance to wear [7]. However, the higher MW hydrogels also exhibited greater friction compared to the lower MW hydrogels.

### 2.9. Discussion of Cartilage Applications

The coefficient of friction in healthy articular joints has been reported to range from 0.002 to 0.01 [20], values that are much lower than the friction measured in this study. Notably, the results are not directly comparable to cartilage friction measurements due to differences in testing conditions. The selection of stainless steel as an opposing surface and deionized water as a lubricating fluid was intended to provide a highly repeatable test for the purpose of statistical comparison between hydrogel variants, which was the primary goal of this study. Using cartilage specimens as an opposing surface and artificial synovial fluid for lubrication would be more representative of physiological conditions, but would lead to less precise comparisons due to the high variability in material properties and morphometry of biological specimens. Previous experiments analyzing the friction of PVA hydrogels when tested against cartilage demonstrated much lower coefficients of friction [23], and similar friction compared to articular fibrocartilage in direct comparisons [22]. For further contextualization, articular cartilage has demonstrated a mean COF of 0.18–0.22 in prior studies under testing conditions similar to the current study (pin-on-plate modality with saline as the lubricating fluid) [7,24].

For the application of cartilage replacement, it may be ideal to utilize a biomimetic material stiffness. A material with an insufficient amount of compliance would not provide sufficient stress dissipation, which could lead to further degeneration or osteoarthritis in the joint. If the chosen material is too soft, it is more likely to have insufficient durability and fail. Overall, the hydrogels exhibited similar compressive moduli to cartilage, considering that the experiments used to derive cartilage moduli used varied testing conditions and strain magnitudes. The low-strain and high-strain PVA hydrogel moduli were similar to the minimum and maximum cartilage moduli, respectively. Depending primarily on annealing temperature, the PVA hydrogels matched or exceeded the tensile moduli of cartilage. The tensile strength of the PVA hydrogel variants was within the range of the articular cartilage data.

### 2.10. Study Limitations

Applying the freeze–thaw treatment to PVA hydrogels produces ice crystal formation. This results in numerous smaller disordered polymer crystallites, close contact of molecular chains, and enlarged pores [7]. Annealing causes the restructuring of disordered crystallites to form larger and more organized crystals, reducing water uptake [7]. This reorganization may have led to the mutual inhibition of the molecular arrangement of the gel, resulting in reduced strength and stiffness.

One limitation of this study is that the testing protocols used on the hydrogels were different than the protocols used in prior tests on articular cartilage. Therefore, the published cartilage data used for comparison represent a general target range for PVA hydrogel properties but were not suitable for direct statistical comparison. The hydrogels synthesized in this study do not replicate the ideal structure and properties of cartilage. Specifically, the PVA hydrogels were homogeneous and isotropic, whereas articular cartilage is heterogeneous and anisotropic. The mechanical testing conditions in this study did not replicate the dynamic loading of joints in vivo. Additionally, the mutual inhibition of freeze–thaw and annealing treatments could be more thoroughly explored for the purpose of treatment optimization. DSC analysis provided insight into the structural changes imposed by annealing the hydrogels in this study. However, DSC did not provide a comprehensive analysis of the material structures, given that the substantial differences in mechanical properties between PVA hydrogel variants were not reflected in crystallinity data derived from DSC. Mechanical properties may have been affected by the organization and distribution of the crystallites, whereas DSC measures only the overall crystallinity. Future studies investigating the degree of cross-linking and the density of the polymer chains of each hydrogel may further elucidate more specific structural changes of the hydrogels.

## 3. Conclusions

Theorizing that the ideal material for cartilage replacement would exhibit high strength and durability, biomimetic stiffness, and low friction, there was no single hydrogel variant that idealized all of these properties. The (10% PVA, 146 kDa 165 °C) hydrogel may have exhibited the best balance of properties due to its high tensile strength, comparably low wear, and a similar tensile modulus to cartilage. However, PVA hydrogels with lower annealing temperatures better matched the compressive modulus of cartilage, and lower MW hydrogels exhibited lower friction.

Utilizing PVA hydrogels for cartilage replacement would require integration of the material with the native tissue. However, PVA hydrogels are highly hydrophilic, which makes them resistant to protein adsorption and cell adhesion [25]. Therefore, solutions for cartilage replacement using PVA hydrogels will likely require composite materials such as a substrate material to anchor the hydrogel to subchondral bone and/or co-polymer blends to promote hydrogel–tissue integration. Further development and testing will be required to create a cartilage replacement solution. In particular, in vivo testing will be critical to assess the safety and efficacy of cartilage replacement or repair using PVA hydrogels, including assessments of the impact of the hydrogels on the tissues in the joint.

## 4. Materials and Methods

### 4.1. PVA Hydrogel Specimen Preparation

PVA (MW of 89,000–98,000 (89K) & 146,000–186,000 (146K), 99+% hydrolyzed, Sigma Aldrich, St. Louis, MO, USA) was mixed with deionized (DI) water in a polymer concentration of 10% or 20% PVA by mass and heated to 98 °C while being stirred for two hours. PVA solutions were poured into rectangular molds with a 2 mm thickness. The molded PVA solutions were physically cross-linked to form hydrogel sheets by using six freeze–thaw cycles (12 h at −18 °C, two hours at 20 °C). After freeze–thaw cycling, the gels were removed from their molds and dried in an oven for four days at 60 °C. This drying procedure was sufficient to show less than 1% mass change in a 24 h period. The hydrogels were placed between two clamped perforated sheets to prevent the PVA hydrogels from curling during drying. Dried PVA hydrogels were then annealed for one hour at a temperature of either 120 °C, 135 °C, 150 °C, or 165 °C. This was a slow treatment process that lasted for four hours. After annealing the hydrogels, they were rehydrated in DI water for at least 24 h.

### 4.2. Polymer Weight Percent

PVA hydrogel specimens were weighed in the fully dried state after annealing and weighed again following rehydration and blotting to remove excess fluid from the surface. Using a sample size of *n* = 1 per group, the polymer weight percent (*wt%*) for each sample was calculated using Equation (1), where *W_D_* represented the dry hydrogel weight (post-annealing) and *W_W_* represented the wet weight (rehydrated) [22].

(1) $$wt\%=\frac{W_{D}}{W_{W}}\times 100\%$$

### 4.3. Unconfined Compression

Unconfined compression tests were performed on cylindrical specimens with a 2 mm diameter extracted via biopsy punch from PVA hydrogel sheets. A sample size of *n* = 5 was used for each group. Samples were mounted on a hydraulic testing machine (Model 370.02, MTS Systems Corporation, Eden Prairie, MN, USA) between two parallel platens. Each sample was loaded to 44% compressive strain at a strain rate of 0.005 s^−1^. Tangent moduli were calculated using localized linear regressions at a low-strain range of 0–4% and a high-strain range of 40–44%, a testing procedure that has been used on similar hydrogels to approximate the physiological range of tissue strains [26].

### 4.4. Tensile Testing

Although cartilage is primarily loaded in compression and shear, this loading produces tensile stresses due to Poisson’s effect. Therefore, tensile properties are important to characterize for the application of cartilage replacement. A sample size of *n* = 5 dog-bone shapes (ISO 527-2 [27]) was cut with an approximate width and thickness of 3.50 mm and 1.50 mm, respectively. Prior to testing, cross-sectional areas were measured via calipers for post hoc stress calculations. Graphite powder was applied to the top surface of each dog-bone specimen to provide fiducial texture for local strain measurement of the specimen’s central region using digital image correlation. A 0.05 N pre-load (approximately 1 kPa) was applied to each specimen, followed by 20 cycles of preconditioning to 4% strain. The specimens were then loaded until failure at a strain rate of 0.08 s^−1^. The ultimate strength for each sample was determined as the maximum stress each sample exhibited. The elastic modulus of each sample was defined as the slope of the linear elastic region of each generated stress–strain curve.

### 4.5. Cyclic Wear Testing

**Figure 10:**
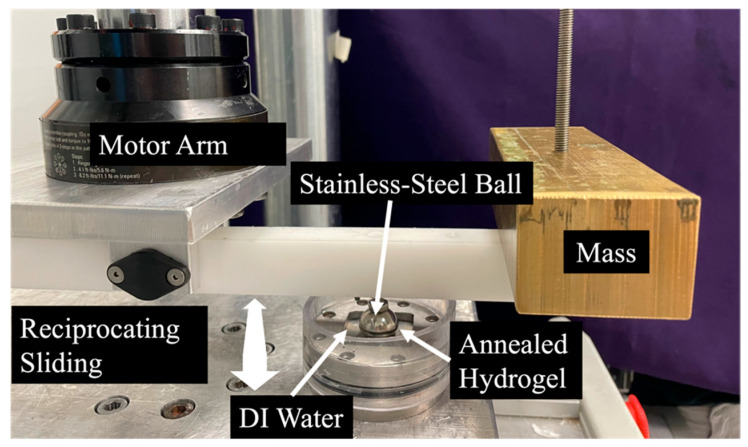
Photograph of the wear testing apparatus.

Three hydrogel variants were selected for wear testing. The 20% PVA, 146 kDa hydrogel annealed at 165 °C was chosen since it was hypothesized that having a greater MW, greater polymer concentration, and the greatest annealing temperature had the greatest probability to be the most durable. Additionally, the 10% 146 kDa and 20% 89 kDa PVA hydrogels (also annealed at 165 °C) were selected to observe the potential effects of MW and polymer concentration. A sample size of *n* = 3 tests was conducted per group. Wear and coefficient of friction (COF) of the PVA hydrogels were found by testing the specimens against the bearing surface of a stainless-steel sphere with a diameter of 19 mm in a reciprocal sliding test. The specimens were clamped onto a torsional hydraulic testing machine at an offset of 10 cm from the center of rotation such that the rotational motion of the actuator resulted in an arc path for the stainless-steel sphere on the fixed hydrogel (Figure 10). A mass was attached to the loading arm connected to the stainless-steel sphere, such that a constant contact force was applied, resulting in an average contact pressure of 0.5 MPa. The samples were immersed in DI water to provide lubrication and maintain hydration.

The sphere was drawn back and forth in a 4.5-degree arc across the surface of each PVA hydrogel specimen, inducing normal and transverse forces that were measured using an axial and torsional load cell (Model 661.19H-03, MTS, Eden Prairie, MN, USA). This was conducted for 0.5 million cycles in each direction at a frequency of 3 Hz. The COF was calculated as the absolute value of the transverse force, taken at the peak of each cycle, divided by the normal force. Reported values are averages of the COF throughout the whole test. Wear depth was determined as the difference in mean hydrogel thickness before and after testing, measured via micrometer in five locations within the contact patch.

### 4.6. Differential Scanning Calorimetry (DSC)

**Figure 11:**
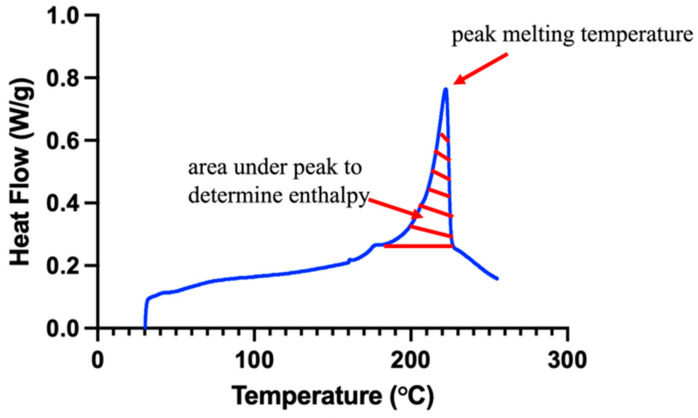
Representative DSC curve (blue) demonstrating the area for enthalpy calculation (red hatch pattern).

Thermogravimetric analysis (TGA) was completed to determine the upper temperature threshold for performing DSC measurements. DSC measurements were performed by using a modulated differential scanning calorimeter (Discovery DSC 2500, TA Instruments, New Castle, DE, USA). A range of 30–300 °C was used at a heating/cooling rate of 5 °C/min to evaluate the peak crystallization and peak melting enthalpies [28]. A 4 mm biopsy punch was used to form *n* = 3 samples per group, with each sample having a mass of 9.0 ± 0.4 mg. The percent crystallinity, $\bar{X_{c}}$, of each sample was calculated using the following equation:

(2) $$\bar{X_{c}}=\;\frac{∆H_{m}}{\Delta H_{ref}}\times 100\%$$

where $∆H_{m}$ represents each sample’s crystallization enthalpy and $∆H_{ref}$ is the reference enthalpy of PVA (taken to be 138.60 J/g [29]). $∆H_{m}$ was found by integrating the area under the melting peak from each specimen’s DSC curve (Figure 11).

### 4.7. Statistical Analysis

Compressive, tensile, and DSC results were analyzed using three-way ANOVAs with Tukey’s post hoc tests to determine the interaction effects of MW, polymer concentration, and annealing temperature. The wear depth and COF results were analyzed using a one-way ANOVA with Tukey’s post hoc test. The high-strain compressive modulus dataset satisfied normality and equal variance assumptions. A natural logarithmic transformation was applied to all other datasets in order to meet the normality and equal variance assumptions for three-way and one-way ANOVAs. All statistical tests utilized a significance threshold of α = 0.05 and were conducted using GraphPad Prism 10 (2365 Northside Dr., Suite 560, San Diego, CA 92108, USA).

## Data Availability

The original contributions presented in this study are included in the article. Further inquiries can be directed to the corresponding author.

## Abbreviations

The following abbreviations are used in this manuscript:

PVA	polyvinyl alcohol
kDa	kilodalton
MW	molecular weight
DSC	differential scanning calorimetry

## References

[1] Nickel J., Iwasaki L., Gonzalez Y., Gallo L., Yao H. (2018). Mechanobehavior and Ontogenesis of the Temporomandibular Joint. J. Dent. Res..

[2] Sophia Fox A.J., Bedi A., Rodeo S.A. (2009). The Basic Science of Articular Cartilage: Structure, Composition, and Function. Sports Health.

[3] Mobasheri A., Kalamegam G., Musumeci G., Batt M.E. (2014). Chondrocyte and mesenchymal stem cell-based therapies for cartilage repair in osteoarthritis and related orthopaedic conditions. Maturitas.

[4] Bray J.C., Merrill E.W. (1973). Poly(vinyl alcohol) hydrogels for synthetic articular cartilage material. J. Biomed. Mater. Res..

[5] Kobayashi M., Oka M. (2004). Characterization of a polyvinyl alcohol-hydrogel artificial articular cartilage prepared by injection molding. J. Biomater. Sci. Polym. Ed..

[6] Li H., Wu C., Yu X., Zhang W. (2023). Recent advances of PVA-based hydrogels in cartilage repair application. J. Mater. Res. Technol..

[7] Chen Y., Li J., Lu J., Ding M., Chen Y. (2022). Synthesis and properties of Poly(vinyl alcohol) hydrogels with high strength and toughness. Polym. Test..

[8] Ningrum D.R., Hanif W., Mardhian D.F., Asri L.A.T.W. (2023). In Vitro Biocompatibility of Hydrogel Polyvinyl Alcohol/Moringa oleifera Leaf Extract/Graphene Oxide for Wound Dressing. Polymers.

[9] Zhao J., Tong H., Kirillova A., Koshut W.J., Malek A., Brigham N.C., Becker M.L., Gall K., Wiley B.J. (2022). A Synthetic Hydrogel Composite with a Strength and Wear Resistance Greater than Cartilage. Adv. Funct. Mater..

[10] Long C., Dong Z., Wang K., Yu F., He C., Chen Z.-R. (2023). Molecular weight distribution shape approach for simultaneously enhancing the stiffness, ductility and strength of isotropic semicrystalline polymers based on linear unimodal and bimodal polyethylenes. Polymer.

[11] Heidarnezhad F., Jafari K., Ozbakkaloglu T. (2020). Effect of polymer content and temperature on mechanical properties of lightweight polymer concrete. Constr. Build. Mater..

[12] Yu W., Wang X., Yin X., Ferraris E., Zhang J. (2023). The effects of thermal annealing on the performance of material extrusion 3D printed polymer parts. Mater. Des..

[13] Kabir W., Di Bella C., Choong P.F., O’cOnnell C.D. (2021). Assessment of Native Human Articular Cartilage: A Biomechanical Protocol. Cartilage.

[14] Shepherd D.E., Seedhom B.B. (1999). The ‘instantaneous’ compressive modulus of human articular cartilage in joints of the lower limb. Natl. Libr. Med..

[15] Bartnikowski M., Wellard R.M., Woodruff M., Klein T. (2015). Tailoring Hydrogel Viscoelasticity with Physical and Chemical Crosslinking. Polymers.

[16] Jeznach O., Kołbuk D., Sajkiewicz P. (2018). Injectable hydrogels and nanocomposite hydrogels for cartilage regeneration. J. Biomed. Mater. Res..

[17] van Mow C., Guo X.E. (2002). Mechano-Electrochemical Properties Of Articular Cartilage: Their Inhomogeneities and Anisotropies. Annu. Rev. Biomed. Eng..

[18] Williamson A.K., Chen A.C., Masuda K., Thonar E.J.M.A., Sah R.L. (2003). Tensile mechanical properties of bovine articular cartilage: Variations with growth and relationships to collagen network components. J. Orthop. Res..

[19] Peppas N.A., Merrill E.W. (1976). Differential scanning calorimetry of crystallized PVA hydrogels. J. Appl. Polym. Sci..

[20] Oungoulian S.R., Durney K.M., Jones B.K., Ahmad C.S., Hung C.T., Ateshian G.A. (2015). Wear and damage of articular cartilage with friction against orthopedic implant materials. J. Biomech..

[21] Gonzalez J.S., Alvarez V.A. (2011). The effect of the annealing on the poly(vinyl alcohol) obtained by freezing–thawing. Thermochim. Acta.

[22] Kuiper J.P., Puttlitz C.M., Rawlinson J.E., Dobbs R., Labus K.M. (2022). A mechanical evaluation of polyvinyl alcohol hydrogels for temporomandibular joint disc replacement. Front. Phys..

[23] Kobayashi M., Hyu H.S. (2010). Development and Evaluation of Polyvinyl Alcohol-Hydrogels as an Artificial Atrticular Cartilage for Orthopedic Implants. Materials.

[24] Oliveira A.S., Seidi O., Ribeiro N., Colaço R., Serro A.P. (2019). Tribomechanical Comparison between PVA Hydrogels Obtained Using Different Processing Conditions and Human Cartilage. Materials.

[25] Wu I.-C., Liou J.-W., Yang C.-H., Chen J.-H., Chen K.-Y., Hung C.-H. (2023). Self-assembly of gelatin and collagen in the polyvinyl alcohol substrate and its influence on cell adhesion, proliferation, shape, spreading and differentiation. Front. Bioeng. Biotechnol..

[26] Beek M., Koolstra J., van Ruijven L., van Eijden T. (2000). Three-dimensional finite element analysis of the human temporomandibular joint disc. J. Biomech..

[27] (2025). Plastics—Determination of Tensile Properties.

[28] Song B., Cao Y., Wang L., Shen Y., Qian X. (2023). Properties and Structure of Thermoplastic Polyvinyl Alcohol/Polyamide Sea-Island Fibers. Polymers.

[29] Stammen J.A., Williams S., Ku D.N., Guldberg R.E. (2001). Mechanical properties of a novel PVA hydrogel in shear and unconfined compression. Biomaterials.

